# Clinical features of patients with candidemia in sepsis

**DOI:** 10.1002/jgf2.250

**Published:** 2019-05-17

**Authors:** Akira Komori, Toshikazu Abe, Shigeki Kushimoto, Hiroshi Ogura, Atsushi Shiraishi, Gautam A. Deshpande, Daizoh Saitoh, Seitaro Fujishima, Toshihiko Mayumi, Satoshi Gando, JAAM FORECAST group, Osamu Tasaki, Yasumitsu Mizobata, Hiraku Funakoshi, Toshiro Okuyama, Iwao Yamashita, Toshio Kanai, Yasuo Yamada, Mayuki Aibiki, Keiji Sato, Susumu Yamashita, Kenichi Yoshida, Shunji Kasaoka, Akihide Kon, Hiroshi Rinka, Hiroshi Kato, Hiroshi Okudera, Eichi Narimatsu, Toshifumi Fujiwara, Manabu Sugita, Yasuo Shichinohe, Hajime Nakae, Ryouji Iiduka, Mitsunobu Nakamura, Yuji Murata, Hiroyasu Ishikura, Yasuhiro Myojo, Yasuyuki Tsujita, Kosaku Kinoshita, Hiroyuki Yamaguchi, Toshihiro Sakurai, Satoru Miyatake, Takao Saotome, Susumu Yasuda, Yutaka Umemura, Junichi Sasaki, Yasukazu Shiino, Taka‐aki Nakada, Takehiko Tarui, Toru Hifumi, Yasuhiro Otomo, Joji Kotani, Yuichiro Sakamoto, Shin‐ichiro Shiraishi, Kiyotsugu Takuma, Ryosuke Tsuruta, Akiyoshi Hagiwara, Kazuma Yamakawa, Naoshi Takeyama, Norio Yamashita, Hiroto Ikeda, Yasuaki Mizushima

**Affiliations:** ^1^ Department of General Medicine Juntendo University Bunkyo Japan; ^2^ Health Services Research and Development Center University of Tsukuba Ibaraki Japan; ^3^ Division of Emergency and Critical Care Medicine Tohoku University Graduate School of Medicine Miyagi Japan; ^4^ Department of Traumatology and Acute Critical Medicine Osaka University Graduate School of Medicine Osaka Japan; ^5^ Emergency and Trauma Center Kameda Medical Center Chiba Japan; ^6^ Division of Traumatology Research Institute National Defense Medical College Saitama Japan; ^7^ Center for General Medicine Education Keio University School of Medicine Shinjuku Japan; ^8^ Department of Emergency Medicine School of Medicine University of Occupational and Environmental Health Fukuoka Japan; ^9^ Division of Acute and Critical Care Medicine Hokkaido University Graduate School of Medicine Hokkaido Japan

To the Editor:

Invasive candidiasis remains a critical problem in intensive care units (ICUs) throughout the developed world.^1^ Nonetheless, differentiating between those with and without invasive candidiasis among critically ill ICU patients, especially during the early phase of the clinical course, remains challenging. In these situations, it is helpful to be familiar with both the epidemiology of the disease and the typical clinical characteristics of patients with candidemia complicated by sepsis, one of the most common risk factors for candidemia.^2^ Our aim was to describe the characteristics and clinical features of patients with candidemia admitted to the ICU due to sepsis.

This is a case series analysis from a sepsis substudy of the Focused Outcomes Research in Emergency Care in Acute Respiratory Distress Syndrome, Sepsis and Trauma (FORECAST) study, a multicenter, prospective cohort study of patients with sepsis. FORECAST was conducted in 59 ICUs from January 2016 to March 2017 in Japan.^3^ Adult patients (≥16 years) with severe sepsis or septic shock based on Sepsis‐2 criteria and admitted to a participating ICU were included. Among this population, we selected patients with candidemia diagnosed from blood culture results.

Of 1184 patients with sepsis, fifteen patients with candidemia were identified (Table 1). Baseline characteristics are shown in Table S1. Six patients died prior to discharge from hospital. Among nine survivors, only one patient was able to return home after discharge (Table S2). The most common species among monomicrobial isolates was *C. albicans* (six patients). Three of the six patients with *C. albicans* and three of the nine patients with non–*C. albicans* died prior to discharge. Catheter‐related blood stream infection (CRBSI) was the most commonly identified source of infection (five patients), followed by lung and abdomen (four patients each). Among five patients with CRBSI, four patients were under 60 years old. All patients with CRBSI survived to discharge, while all patients with fungal pneumonia or empyema died.

**Table 1:** Characteristics and outcome of fifteen patients with candidemia complicated by sepsis (n = 15)

Age	Gender	Infection site	Admission source	Shock	Medication	Comorbidities	Antibiotic use before diagnosis	MV	Vasopressor use	SOFA score	APACHE II score	Blood culture		Antifungal drug	Antibiotics	LOS (d)	ICU‐FD (d)	Disposition
67	M	Abdomen	ED	Yes	No	COPD	Yes	Yes	Yes	11	23	*C albicans*	*E faecium E coli*		MEPM, VCM	17	0	Dead
46	M	CRBSI	Ward (transfer)	No	No		Yes	No	No	8	23	*C albicans*		F‐FLCZ		74	8	Transfer
76	M	Lung	Ward (transfer)	Yes	Anticancer drug	Metastatic malignant tumor	Yes	Yes	No	12	37	*C tropicalis*		MCFG	MEPM, VCM	27	0	Dead
45	M	Lung	Ward (transfer)	Yes	Steroid	Connective tissue disease, Peptic ulcer disease, DM	Yes	No	Yes	12	28	*C albicans*	*C striatum*	F‐FLCZ	MEPM, VCM	15	0	Dead
46	M	CRBSI	Ward (transfer)	Yes	No		No	NA	Yes	NA	NA	*C glabrata*		FLCZ		11	23	Transfer
78	M	Abdomen	Ward (transfer)	No	Steroid	Connective tissue disease	No	No	No	3	20	*C glabrata*	*E coli*	MCFG	MEPM	27	8	Transfer
60	M	CRBSI	Ward (transfer)	Yes	No	Cerebrovascular disease, DM, hemiplegia	No	Yes	Yes	17	31	*C tropicalis*	*P mirabilis*		MEPM, VCM	27	15	Transfer
85	W	Abdomen	ED	Yes	Anticoagulant	Dementia	No	No	Yes	8	15	*C parapsilosis*	*Pseudomonas sp*.		CMZ	96	9	Transfer
30	W	CRBSI	Ward (transfer)	No	No		Yes	Yes	Yes	9	30	*C parapsilosis*		F‐FLCZ	CFPM	142	22	Home
62	W	Wound	Ward (transfer)	Yes	No		No	Yes	Yes	11	13	*C glabrata*	*P mirabilis*	VRCZ, MCFG	CTRX, MEPM, VCM, DAP	19	0	Dead
86	M	CRBSI	Ward (transfer)	Yes	No	Cerebrovascular disease, COPD	Yes	No	Yes	NA	23	*C tropicalis*			MEPM	47	23	Transfer
46	W	Abdomen	Ward (transfer)	Yes	No		Yes	Yes	Yes	7	18	*C albicans*			MEPM	165	0	Transfer
75	M	Lung	Ward (transfer)	Yes	No	Malignancy (solid)	Yes	Yes	Yes	8	17	*C glabrata*		L‐AMB	CZOP	149	0	Dead
71	W	Lung	Ward (transfer)	No	No	Cerebrovascular disease, DM, liver disease	Yes	No	Yes	11	32	*C albicans*			PIPC/TAZ	6	0	Dead
71	M	Other site	Ward (transfer)	No	No	DM, malignancy (solid)	Yes	Yes	Yes	11	32	*C albicans*			PIPC/TAZ, CLDM	90	11	Transfer

APACHE II, acute physiology and chronic health evaluation II; CFPM: cefepime; CLDM: clindamycin; CMZ: cefmetazole; COPD: chronic obstructive pulmonary disease; CRBSI: catheter‐related blood stream infection; CTRX: ceftriaxone; CZOP: cefozopran; DAP: daptomycin; DM: diabetes mellitus; ED: emergency department; F‐FLCZ: fosfluconazole; FLCZ: fluconazole; ICU‐FD: ICU‐free days; L‐AMB: liposomal amphotericin B; LOS: length of hospital stay; VRCZ: voriconazole; M: men; MCFG: micafungin; MEPM: meropenem; MV: mechanical ventilation; PIPC/TAZ: piperacillin/tazobactam; SOFA: Sequential Organ Failure Assessment; VCM: vancomycin; W: women.

Other site was not lung, abdomen, urinary tract, soft tissue, central nervous system, osteoarticular, endocardium, wound, and implant device.

The incidence of candidemia among patients with sepsis in the FORECAST study 3 was 1.3%, which was comparable with previous studies^4^ despite significant geographical differences in the Candida species.^5^ Candidemia should always prompt a search for the source despite the possibility of reflecting colonization of an indwelling intravenous catheter. Our results showed differences in patients with candidemia complicated by sepsis. Deep‐seated infections, which lead to secondary candidemia, were associated with high mortality compared to CRBSI. However, underestimation of patients with candidemia is likely as rates of fungal blood culture positivity are lower than for bacteria. Although there is not yet a magic bullet for the diagnosis of this elusive disease, clinical information, such as comorbidities, need for mechanical ventilation, elevated severity score, and low albumin, remains crucially important in providing clues to the diagnosis of candidemia.^1^

In conclusion, this is the first report to provide a detailed description of septic patients with candidemia in ICU in Japan. In terms of nosocomial infections, candidemia from deep‐seated sources had poorer outcomes compared to those with candidemia caused by CRBSI. Early recognition of fungal infection remains key.

## CONFLICT OF INTERESTS

The authors have stated explicity that there are no conflict of interest in connection with this article.

## Investigators of JAAM FORECAST Study Group

Nagasaki University Hospital (Osamu Tasaki); Osaka City University Hospital (Yasumitsu Mizobata); Tokyobay Urayasu Ichikawa Medical Center (Hiraku Funakoshi); Aso Iizuka Hospital (Toshiro Okuyama); Tomei Atsugi Hospital (Iwao Yamashita); Hiratsuka City Hospital (Toshio Kanai); National Hospital Organization Sendai Medical Center (Yasuo Yamada); Ehime University Hospital (Mayuki Aibiki); Okayama University Hospital (Keiji Sato); Tokuyama Central Hospital (Susumu Yamashita); Fukuyama City Hospital (Susumu Yamashita); JA Hiroshima General Hospital (Kenichi Yoshida); Kumamoto University Hospital (Shunji Kasaoka); Hachinohe City Hospital (Akihide Kon); Osaka City General Hospital (Hiroshi Rinka); National Hospital Organization Disaster Medical Center (Hiroshi Kato); University of Toyama (Hiroshi Okudera); Sapporo Medical University (Eichi Narimatsu); Okayama Saiseikai General Hospital (Toshifumi Fujiwara); Juntendo University Nerima Hospital (Manabu Sugita); National Hospital Organization Hokkaido Medical Center (Yasuo Shichinohe); Akita University Hospital (Hajime Nakae); Japanese Red Cross Society Kyoto Daini Hospital (Ryouji Iiduka); Maebashi Red Cross Hospital (Mitsunobu Nakamura); Sendai City Hospital (Yuji Murata); Subaru Health Insurance Society Ota Memorial Hospital (Yoshitake Sato); Fukuoka University Hospital (Hiroyasu Ishikura); Ishikawa Prefectural Central Hospital (Yasuhiro Myojo); Shiga University of Medical Science (Yasuyuki Tsujita); Nihon University School of Medicine (Kosaku Kinoshita); Seirei Yokohama General Hospital (Hiroyuki Yamaguchi); National Hospital Organization Kumamoto Medical Center (Toshihiro Sakurai); Saiseikai Utsunomiya Hospital (Satoru Miyatake); National Hospital Organization Higashi‐Ohmi General Medical Center (Takao Saotome); National Hospital Organization Mito Medical Center (Susumu Yasuda); Tsukuba Medical Center Hospital (Toshikazu Abe); Osaka University Graduate School of Medicine (Hiroshi Ogura, Yutaka Umemura); Kameda Medical Center (Atsushi Shiraishi); Tohoku University Graduate School of Medicine (Shigeki Kushimoto); National Defense Medical College (Daizoh Saitoh); Keio University School of Medicine (Seitaro Fujishima, Junichi Sasaki); University of Occupational and Environmental Health (Toshihiko Mayumi); Kawasaki Medical School (Yasukazu Shiino); Chiba University Graduate School of Medicine (Taka‐aki Nakada); Kyorin University School of Medicine (Takehiko Tarui); Kagawa University Hospital (Toru Hifumi); Tokyo Medical and Dental University (Yasuhiro Otomo); Hyogo College of Medicine (Joji Kotani); Saga University Hospital (Yuichiro Sakamoto); Aizu Chuo Hospital (Shin‐ichiro Shiraishi); Kawasaki Municipal Kawasaki Hospital (Kiyotsugu Takuma); Yamaguchi University Hospital (Ryosuke Tsuruta); Center Hospital of the National Center for Global Health and Medicine (Akiyoshi Hagiwara); Osaka General Medical Center (Kazuma Yamakawa); Aichi Medical University Hospital (Naoshi Takeyama); Kurume University Hospital (Norio Yamashita); Teikyo University School of Medicine (Hiroto Ikeda); Rinku General Medical Center (Yasuaki Mizushima); and Hokkaido University Graduate School of Medicine (Satoshi Gando).
