# Supplementary material for: Clinical features of patients with candidemia in sepsis
Source: J Gen Fam Med. 2019 May 17;20(4):161–3. doi: 10.1002/jgf2.250 (PMC6612772; doi:10.1002/jgf2.250)
Supplement: Supplementary file 1 [file JGF2-20-161-s001.docx]

Supplementary Table1: Characteristics of patients with candidemia complicated by sepsis (n=15)

| Characteristics |  |
| --- | --- |
| Age at admission — Median (IQR) | 67 (46.0-75.5) |
| Male — no. (%) | 10 (66.7) |
| BMI — Median (IQR) | 21 (18.4-24.7) |
| Admission source — no. (%) |  |
| ED | 2 (13.3) |
| Ward (transfer) | 13 (86.7) |
| Coexisting conditions — no. (%) |  |
| Myocardial infarction | 0 |
| Congestive heart failure | 0 |
| Peripheral vascular disease | 0 |
| Cerebrovascular disease | 3 (20.0) |
| Dementia | 1 (6.7) |
| COPD | 2 (13.3) |
| Connective tissue disease | 2 (13.3) |
| Peptic ulcer disease | 1 (6.7) |
| Diabetes mellitus without organ damage | 3 (20.0) |
| Diabetes mellitus with organ damage | 1 (6.7) |
| Chronic kidney disease | 0 |
| Hemiplegia | 1 (6.7) |
| Malignancy (solid) | 2 (13.3) |
| Malignancy (blood) | 0 |
| Metastatic tumor | 1 (6.7) |
| Mild liver disease | 0 |
| Moderate to severe liver disease | 1 (6.7) |
| AIDS | 0 |
| Charlson Comorbidity Index — Median (IQR) | 1 (0-3) |
| Medication — no. (%) |  |
| Steroids | 2 (13.3) |
| Immunosuppressants | 0 |
| Anticancer drug | 1 (6.7) |
| Radiation therapy | 0 |
| Anticoagulant | 1 (6.7) |
| Antibiotics use before diagnosis — no. (%) | 10 (66.7) |
| Septic shock — no. (%) | 10 (66.7) |
| APACHE II score — Median (IQR) | 23 (19-31) |
| SOFA score — Median (IQR) | 11 (8-11) |
| Laboratory Data — Median (IQR) |  |
| WBC (/µL) | 10150 (5300-15225) |
| Platelet (×10^4/µL) | 6.6 (3.6-12.5) |
| Lactate (mmol/L) | 2.9 (2.0-3.4) |
| CRP (mg/dL) | 15.5 (12.1-19.4) |
| Procalcitonin (mg/dL) | 3.2 (1.1-17.3) |
| Albumin (mg/dL) | 1.7 (1.5-1.9) |

IQR: interquartile range, BMI: body mass index, ED: emergency department, COPD: chronic obstructive pulmonary disease, AIDS: acquired immunodeficiency syndrome, APACHE Ⅱ: acute physiology and chronic health evaluation Ⅱ, SOFA: sequential organ failure assessment, WBC: white blood cell, CRP: c-reactive protein.
