# Supplementary material for: Clinical features of patients with candidemia in sepsis
Source: J Gen Fam Med. 2019 May 17;20(4):161–3. doi: 10.1002/jgf2.250 (PMC6612772; doi:10.1002/jgf2.250)
Supplement: Supplementary file 2 [file JGF2-20-161-s002.docx]

Supplementary Table 2．Outcome and disposition of patients with candidemia complicated by sepsis (n=15)

| Outcomes | Units |  |
| --- | --- | --- |
| In-hospital mortality | No./total (%) | 6 (40.0) |
| In-hospital mortality with shock (n=10) | No./total (%) | 5 (50.0) |
| Survivor dispositions |  |  |
| Home | No./total (%) | 1 (11.1) |
| Transfer | No./total (%) | 8 (88.9) |
| ICU-free days | Median (IQR) | 8 (0-13) |
| Ventilator-free days | Median (IQR) | 4 (0-20.5) |
| Length of hospital stay | Median (IQR) | 27 (18-93) |

IQR: interquartile range, ICU: intensive care unit
